# Prevalence of intrafamilial child maltreatment in a school in Quito, Ecuador

**DOI:** 10.3389/fped.2026.1907946

**Published:** 2026-09-14

**Authors:** María José Carrera Yánez, Kenneth Xavier Galarza Rivadeneira

**Affiliations:** Academic Area of Arts and Humanities, Psychology Program, Universidad Tecnológica Israel, Quito, Ecuador

**Keywords:** child maltreatment, domestic violence, family relations, physical abuse, psychological abuse, neglect, students, mental health

## Abstract

**Introduction:**

Intrafamilial child maltreatment poses a serious risk to children's and adolescents' physical, psychological, and sexual integrity. This study analyzed the prevalence of this phenomenon among students of the Enrique Proaño Educational Institution in Quito, Ecuador, during the 2024–2025 period.

**Methods:**

A quantitative, descriptive design was used to assess the dimensions of physical, psychological, and sexual abuse, as well as neglect, considering the involvement of maternal and paternal figures.

**Results:**

Physical abuse showed the highest prevalence among the maltreatment types assessed, followed by moderate levels of neglect. Psychological abuse occurred less frequently but remains a significant concern, and sexual abuse, although low in prevalence, was still present. The maternal figure was more frequently associated with neglect, whereas the paternal figure was more often linked to physical abuse.

**Discussion:**

These patterns highlight the need for prevention and intervention strategies tailored to both the type of maltreatment and the parental figure involved.

## Introduction

Intrafamilial child maltreatment represents a latent risk to the physical, psychological, and sexual integrity of children and adolescents, exposing them to circumstances that seriously violate their rights. These acts are perpetrated by parents, caregivers, or guardians responsible for their care, thereby affecting their overall development. “Child maltreatment is a problem that occurs across different spheres of society, in which rights such as the physical, psychological, and sexual integrity of children are violated” (1). The study conducted at the Daniel Enrique Proaño Educational Unit, located in Quito, Ecuador, during the 2024–2025 period, primarily aimed to determine the prevalence of this phenomenon. The research addressed various forms of maltreatment, including physical, psychological, sexual, and neglectful abuse. Neglect is defined as the failure to meet and provide for children's psychological, physical, and protective needs, “(…) or to obtain medical care, birth registration, or other services, even when caregivers have the means, knowledge, and access to do so” (2). A quantitative descriptive approach was employed, as it was considered most appropriate for the study. “The information provided by descriptive research must be truthful, precise, and systematic. Inferences about the phenomenon should be avoided; the focus lies on observable and verifiable characteristics” (3). For data processing and analysis, statistical tools such as the Statistical Package for the Social Sciences (SPSS) and Microsoft Excel were utilized. These tools facilitated the organization and interpretation of the data, understood as “the practice of integrating and consolidating data sources into a single dataset, with the ultimate aim of systematically providing readers with access to information” (4). The results were presented in descriptive tables, offering a clear and structured overview of the data obtained. These tables include the findings of the present study as well as direct comparisons with previous research (5). This approach allows for the contextualization of the results within a broader framework, enabling the identification of patterns, similarities, and discrepancies with earlier studies. By incorporating the data into this comparative framework, a deeper understanding of the implications and significance of the findings is achieved in relation to the existing body of knowledge in the field. Furthermore (6), significant differences in types of maltreatment were examined with respect to maternal and paternal figures, revealing patterns and associated risk factors. Characteristics that may increase the risk of child maltreatment by parents include: “difficulty in establishing an emotional bond with the newborn; failure to care for the child; having experienced maltreatment during one's own childhood; lack of knowledge about child development; or holding unrealistic expectations” (7). The conclusions derived from this research highlight the urgent need to implement effective intervention strategies aimed at protecting the violated rights of children and adolescents. “It is important to point out that these institutions have established response pathways for cases involving children whose rights have been violated” (8). In addressing minors affected by intrafamilial maltreatment, these institutions aim to strengthen early detection systems, comprehensive care, and psychosocial support for victims.

## Methodology

A quantitative approach was adopted, allowing for the collection of statistical data through an instrument that effectively evaluates the proposed variable. “It is used to consolidate beliefs (formulated logically in a theory or theoretical framework) and to accurately establish patterns of behavior within a population” (9). This approach facilitated subsequent analysis of data related to intrafamilial child maltreatment.

The study was conducted with a population of 233 students enrolled in upper Basic General Education (8th, 9th, and 10th grades) at the Daniel Enrique Proaño Educational Unit, located in La Colmena parish, Quito canton, Pichincha province, during the 2024–2025 academic period.

A non-probability sampling method was employed, as the total population of 233 students was considered sufficient for the study. “In non-probability samples, the selection of elements does not depend on probability but on causes related to the characteristics of the research or the researcher's objectives” (9). The study included the entire population of 8th, 9th, and 10th grade students in upper Basic General Education, ensuring proper application, review, and analysis of the instruments.

The Intrafamilial Child Maltreatment Questionnaire was used as the primary data collection instrument. By definition, it is “a valuable technique in research across different fields, allowing researchers to gather information from a large number of individuals in a short period of time, making it an efficient and economical tool” (10). The questionnaire used in this study was developed by the research team based on the studýs objectives and the conceptual dimensions related to intrafamilial child maltreatment. In order to assess the content validity of the instrument, the questionnaire underwent an expert review process, during which experts evaluated the relevance, clarity, consistency and appropriateness of the items for the target population. Subsequently, a pilot study was conducted in an Ecuadorian population similar to the study people in which the instrument would be administered. This phase of instrument development and validation made it possible to assess respondent´s understanding of the items, identify potential difficulties in completing the questionnaire, and make the necessary adjustments before the official administration. The study obtained a Cronbach's alpha coefficient of 0.93, indicating excellent internal consistency. The questionnaire consisted of 66 items, structured to identify family figures and based on a Likert-type scale. “This instrument includes items designed to assess attitudinal positions using an ordinal rating scale, which includes a neutral point and options indicating disagreement and agreement, with numerical values ranging from 1 to 5” (11). The response categories were defined as follows: Never (1), Almost never (2), Sometimes (3), Almost always (4), Always (5).

This instrument evaluates the dimensions of physical abuse, emotional abuse, neglect, and sexual abuse, with a maximum score of 330 points and a minimum of 66. “The first requirement is the collection of relevant data, its processing, and subsequent analysis. The data collected are measurements that usually derive from real observations obtained through sampling a population” (12).

The collected data were entered into a database for statistical analysis, using Microsoft Excel to graphically represent the results. Coding was performed directly in SPSS for detailed statistical analysis, including the use of contingency tables. The validity of the instrument was ensured by evaluating its ability to measure the research variable, following content and construct validity criteria, with an expected value equal to or greater than 0.80.

The internal consistency of the instrument was assessed to determine the reliability of the scores obtained. “It allows determining whether the scores of the evaluated sample are reliable from the perspective of internal consistency, providing evidence of measurement quality” (13). Reliability was evaluated using Cronbach's alpha, which, according to International Business Machines, “allows the analysis of the properties of measurement scales and their components” (14). Additionally, the reliability of the dimensions of physical abuse, emotional abuse, neglect, and sexual abuse was calculated using Spearman-Brown coefficients, yielding high values.

A normalization process (development of norms) was conducted using Z-scores to analyze the distance of each score from the mean. The mean of Z is always 0, and its standard deviation is always 1. For example, a Z-score of −1.90 indicates that subjects with a raw score of 8 (from a total of 30 participants in the pilot test) are located 1.90 standard deviations below the sample mean, justifying the elimination of duplicated results with score X. This raw value falls below the mean because its standardized score is negative. All subjects with raw scores below the mean will have negative Z-scores, while those above the mean will have positive Z-scores. This process enabled the representation of the distribution of scores across the different dimensions of intrafamilial child maltreatment and ensured the applicability of the instrument within the local context.

Ethical considerations are a cross-cutting theme throughout the research process, particularly in studies relating to domestic child abuse, given the sensitivity of the subject matter and the vulnerable nature of the participating population. In this regard, the following details how the main ethical aspects were addressed during the course of the research.

Firstly, it was ensured that the participation of children and adolescents was entirely voluntary, with the informed consent of their legal guardians and, in parallel, the assent of the participants themselves, appropriate to their level of development and understanding. In cases where there were limitations on expressing such consent verbally or in writing, adapted strategies were implemented to validate their willingness to participate, ensuring respect for their wishes and specific circumstances.

Secondly, the role of children and adolescents as active participants in the research process was clearly defined, recognizing their ability to express experiences, perceptions and meanings relating to the issue under study. Furthermore, efforts were made to ensure that the results obtained were communicated in a comprehensible and respectful manner, promoting their inclusion and recognition within the process.

In addition, specific measures were implemented to ensure the comprehensive protection of their rights, such as maintaining the confidentiality of information, safeguarding their identity through the use of codes or pseudonyms, and applying protocols for action in the event of identifying situations of risk or rights violations, referring cases to the relevant authorities where necessary.

Finally, a rights-based approach was adopted throughout the document, using inclusive language that recognizes children and adolescents as rights-holders and social actors. In this regard, the use of terms that might be reductive or that obscure their agency was avoided, promoting a respectful, dignified and contextualized view of their realities.

## Results

### Analysis of the dimensions of intrafamilial child maltreatment

Through the application of the intrafamilial child abuse questionnaire, it was possible to identify the following data regarding the general levels of intrafamilial child maltreatment among the 233 surveyed students, who represent 100% of the sample. These results were also analyzed specifically by dividing the population into two groups (fathers and mothers).

Table 1, which presents the overall intrafamilial child maltreatment, shows that there is a predominance of a medium level, representing 48.96% of the population. Additionally, 34.74% of students report a high level of intrafamilial child maltreatment, which reflects a concerning issue within the population, particularly in relation to parenting styles and the emotional education of parents.

**Table 1:** Intrafamilial child abuse general test.

Maltreatment	Frequency	Percentage
Low	38	16.30
Medium	114	48.96
High	81	34.74
Total	233	100.00

Within the data indicate that a large proportion of the population experiences some form of maltreatment, while only 16.30% report a low level.

Within this population, an analysis was conducted of both parental figures in relation to intrafamilial child maltreatment, as shown in Table 2. Regarding the maternal role, 56.23% of students perceive a medium level, followed by 38.64% reporting a low level and 5.13% a high level, suggesting a higher incidence of moderate maltreatment associated with the mother. In comparison, the paternal figure shows a medium level in 55.35% of students, followed by 38.60% reporting low levels and 6.05% high levels.

**Table 2:** General intra-family child abuse by mother and father.

Maternal abuse	Frequency	Percentage	Parental abuse	Frequency	Percentage
Low	90	38.64	Low	90	38,60
Medium	131	56.23	Medium	129	55,35
High	12	5.13	High	14	6,05
Total	233	100.00	Total	233	100,00

In both cases, most of the population falls within medium levels, indicating that there is no highly significant distinction between the two parental figures from a general perspective.

Table 3 presents the overall physical abuse results, which reveal concerning levels. Specifically, 70.5% of students fall within the medium level, representing a large portion of the population. This suggests that within the educational system, this issue may not have been addressed with sufficient rigor or adequately discussed. Additionally, 4.0% of students present a high level of physical abuse, while the remaining 25.5% fall within a low level.

**Table 3:** General physical abuse.

Categorization	Frequency	Percentage
Low	59	25.5
Medium	163	70.5
High	11	4.0
Total	233	100.0

In the data reveal the presence of physical abuse at various levels within the study population, with intermediate categories being the most common, suggesting a need to strengthen measures for prevention, detection and timely intervention within the educational context.

Table 4 shows that the overall prevalence of psychological abuse is 52.7% at a medium level. This indicates that the majority of children experience some form of psychological abuse, often manifested through verbal and symbolic actions that leave a lasting impact on students. This reflects a significant proportion of individuals being raised within environments that involve some degree of abuse. Considering that 1.6% of students report a high level, the cases are concerning. Meanwhile, 45.7% indicate a low level, which, although it may appear favorable in statistical terms, does not necessarily imply the absence of maltreatment, but rather the presence of minimal levels.

**Table 4:** General psychological abuse.

Categorization	Frequency	Percentage
Low	106	45.7
Medium	123	52.7
High	4	1.6
Total	233	100.0

The results indicate the presence of psychological abuse within the study population at various levels, with a predominance of intermediate categories, highlighting the importance of implementing intervention and support strategies to address this issue within educational and family settings.

In Table 5, neglect—understood as the lack of attention to the needs of children—shows an alarming prevalence, with 60.4% at the medium level and 13.6% at the high level. This indicates that approximately 74% of this population is affected by some form of neglect within their family environment. This suggests that parental figures either are unwilling or unable to adequately meet the full range of children's needs, resulting in a suboptimal environment for their growth and development.

**Table 5:** General negligence.

Categorization	Frequency	Percentage
Low	61	26.0
Medium	140	60.4
High	32	13.6
Total	233	100.0

The results in contrast, only 26.0% fall within the low level, indicating that a relatively small portion of the population has their needs adequately met.

In Table 6, regarding sexual abuse, the results show that 0.46% of students report a high level, 4.28% a medium level, and 95.26% fall within the low level. Although the values obtained from the survey are low, it is important to note that these data may be underreported due to the sensitive and stigmatized nature of sexual abuse.

**Table 6:** Sexual abuse.

Categorization	Frequency	Percentage
None	222	95.26
Medium	10	4.28
High	1	0.46
Total	233	100.00

This may hinder victims from disclosing their experiences, as fear of social disapproval and feelings of shame can prevent them from reporting the situation, contributing to the invisibility of this type of maltreatment.

Table 7 presents the data regarding both parental figures for each indicator of the studied variable. Concerning the mother, the statistical results indicate that 25.35% of students report a high level of physical abuse, 22.74% a medium level, and 51.91% a low level.

**Table 7:** Child maltreatment by type of abuse according to parental figures.

Mother	Physical Abuse		Emotional Abuse		Neglect	
Categorization	Frequency	Percentage	Frequency	Percentage	Frequency	Percentage
Low	121	51.91	141	60.58	162	69.50
Medium	59	25.35	54	23.10	71	30.50
High	53	22.74	38	16.33	0	0.00
	233	100.00	233	100.00	233	100.00

Father	Physical Abuse		Emotional Abuse		Neglect	
Categorization	Frequency	Percentage	Frequency	Percentage	Frequency	Percentage
Low	111	47.68	135	57.94	158	67.79
Medium	63	27.01	61	26.22	75	32.21
High	59	25.31	37	15.84	0	0.00
	233	100.00	233	100.00	233	100.00

This the results reveal differences in the prevalence of various forms of abuse depending on the type of caregiver, with variations observed across low, medium and high levels in the indicators assessed, highlighting the importance of taking a holistic view of the family context in analysis, prevention and intervention processes.

Regarding psychological abuse, 16.33% of students report a high level, 23.10% a medium level, and 60.58% a low level. In terms of maternal neglect, 30.50% present a medium level, while 69.50% report a low level. In relation to the father, the data indicate that 25.31% of students report a high level of physical abuse, 27.01% a medium level, and 47.68% a low level. Regarding psychological abuse, 15.84% of students indicate a high level and 26.22% a medium level.

With respect to neglect, 32.21% of students report a medium level and 67.79% a low level, suggesting the possible presence of deficiencies in the child’s immediate environment.

## Discussion

Upon completion of the research on intrafamilial child maltreatment among upper Basic General Education students at the Daniel Enrique Proaño Educational Unit, it was found that the present study yields result similar to those reported (15) in their study titled “Impact of child maltreatment on the prevalence of mental disorders in Chilean children and adolescents.” The authors concluded that psychological child maltreatment affects 54.4% of the population, highlighting a clear need for intervention. These findings closely resemble those of the present study, which reports a percentage of 52.7% of students. Additionally (15), reported a prevalence of neglect at 32.2%, which differs considerably from the 60.4% found in the present study. Similarly, physical abuse was reported at 7.0% in their study, contrasting sharply with the 70.5% identified in this research. Regarding sexual abuse, they reported a prevalence of 6.4%, which is relatively similar to the 4.8% observed in this study.

On the other hand, with respect to the primary family figure responsible for maltreatment, in their study “Intrafamilial Child Maltreatment in Basic General Education Students,” concluded that the mother is more frequently the source of maltreatment, accounting for 91.04%. They note that the maternal role is influenced by both personal and social factors, often involving corrective practices of a physical and emotional nature. In contrast, the present study indicates that there is no single predominant parental figure responsible for maltreatment (16). Both fathers and mothers exhibit similar levels of medium and high maltreatment, with a combined prevalence of 57.4%. This reflects that child-rearing and education are largely family-based processes influenced by both parental figures, who act as primary agents in the transmission of values and norms.

In the study (17), 42.9% of participants reported experiencing psychological maltreatment and 35.9% physical maltreatment, while only 29.1% identified themselves as victims of child maltreatment overall. In contrast, the present study shows that 4.0% of students report a high level of physical abuse, 1.6% a high level of psychological abuse, and 13.6% a high level of neglect. These findings reflect different forms and degrees of impact among the surveyed population. Both studies converge on the critical importance of emotional abuse as a key factor affecting children's well-being.

Similarly, in their study “Consequences of Child Maltreatment in a Hospital in Maracaibo, Venezuela,” found that 30.0% of participants reported psychological abuse and 34.54% physical abuse, while 26.36% reported neglect and 9.09% sexual abuse (18). In contrast, the present study shows that 70.5% of students fall within a medium level of physical abuse, 52.7% within a medium level of psychological abuse, and 60.4% within a medium level of neglect. These results reflect variations in the prevalence and forms of maltreatment among different populations. Both studies highlight physical abuse as one of the most prevalent forms of disciplinary practice.

Overall, the findings of this study, in comparison with previous research, present both similarities and discrepancies, which may be attributed to various contextual and methodological factors influencing the results.

The present study identified four types of intrafamilial child maltreatment, primarily perpetrated by parents: physical abuse, emotional abuse, neglect, and sexual abuse. The results show a notably high prevalence of physical abuse compared to other forms of maltreatment. This high prevalence may be associated with the difficulty's parents face in managing and expressing negative emotions in an assertive manner. A lack of emotional regulation skills may lead parents to resort to physical abuse as an inappropriate response to conflict situations with their children.

Parents who struggle to regulate unfavorable emotions may become trapped in cycles of harmful behavior, in which physical abuse becomes a recurring response to internal frustration and conflict dynamics within the household. This finding highlights the need for family-level interventions that provide parents with tools and support to develop emotional regulation and positive communication skills. Psychoeducational programs and parental support initiatives could play a crucial role in preventing physical abuse and promoting healthier family environments.

Students reported varying levels of neglect, generally at a medium level, indicating a concerning deficiency in meeting basic needs. Educational neglect was particularly prominent, associated with a lack of parental involvement in children's learning processes and academic performance. Physical neglect was reflected in inadequate nutrition, clothing, personal care, and supervision. Emotional neglect manifested as a lack of affection and emotional support, with children often being ignored or dismissed. Medical neglect was observed in the lack of attention to health needs, including delays in treatment and insufficient medical care, posing risks to children's well-being.

In contrast, psychological abuse, although less prevalent than neglect, represents a significant dimension of maltreatment. It is expressed through the inability to provide a stable emotional environment and includes behaviors such as insults, intimidation, indifference, rejection, lack of affection, and verbal aggression. These experiences not only affect children's emotional well-being but also have a profound impact on their self-esteem, resilience, ability to form healthy relationships, and overall psychological development. It is essential that child protection policies and programs address psychological abuse to ensure a comprehensive response to the needs of affected children.

Regarding sexual abuse, although its prevalence among the participants was low, it remains a critical issue. The identified cases indicate that this problem persists and can have severe consequences for children. It is possible that such incidents remain underreported due to the conditions under which they occur and the difficulties victims face in disclosing them. Social stigma surrounding these experiences may lead children and adolescents to feel unable or unwilling to express their distress.

The findings of this study indicate that both fathers and mothers play significant roles in cases of child maltreatment; however, distinct patterns emerge regarding the types of abuse committed. The maternal figure is more frequently associated with neglect-related behaviors, such as lack of attention to personal hygiene and limited involvement in children's academic responsibilities. This suggests that maternal neglect may be linked to insufficient supervision and support in children's development.

In contrast, the paternal figure is more strongly associated with physical abuse, often involving corrective practices such as hitting, slapping, or the use of objects as punishment. These findings suggest that while mothers may be more involved in neglect-related forms of maltreatment, fathers are more likely to engage in physically abusive disciplinary practices.

These differences in patterns of maltreatment highlight the need for targeted approaches in child protection interventions and policies. Prevention and support programs should address negligent and abusive behaviors in differentiated ways, considering the characteristics of perpetrators and the specific type of maltreatment. Furthermore, it is essential to promote awareness and education regarding the impact of these behaviors on child development, as well as to provide resources that support parents and caregivers in adopting healthier and more constructive practices.

This study underscores the importance of a comprehensive intervention approach that not only identifies and addresses abusive behaviors but also supports families in creating safe and nurturing environments for the well-being and optimal development of children.

The study has some limitations; among them, the research was conducted at a single educational institution in Quito-Ecuador, which limits the generalizability of the results to other institutions, regions and populations with different sociodemographic characteristics. Furthermore, because of the studýs cross-sectional design, the findings allow us to describe the prevalence of intrafamilial child maltreatment at the time of the assessment, buy they do not allow us to establish causal relationships. It is important to emphasize that the information was obtained through a self-report questionnaire; therebefore, the presence of response bias cannot be ruled out. Because domestic child abuse is a sensitive topic, several participants may have had difficulty disclosing certain experiences, which could lead to underreporting. Although the instrument was developed by the researchers, it underwent validation through expert judgment and was subsequently evaluated in a pilot study; similar findings in future research could strengthen the evidence of validity through studies with larger, more representative simples from different educational contexts and regions of the country. It is important to note that these limitations do not invalidate the findings, but they must be considered when interpreting their scope and generalizability.

## Data Availability

The raw data supporting the conclusions of this article will be made available by the authors, without undue reservation.
